# Pandemic upon Pandemic: Middle-Aged and Older Men Who Have Sex with Men Living with HIV Coping and Thriving during the Peak of COVID-19

**DOI:** 10.3390/ijerph20115979

**Published:** 2023-05-28

**Authors:** Sherry Bell, Brandon Ranuschio, John M. Waldron, Lianne Barnes, Nadia Sheik-Yosef, Esmeralda Villalobos, Janelle Wackens, Renato M. Liboro

**Affiliations:** 1 Department of Psychology, University of Nevada, Las Vegas, NV 89154, USA; bells12@unlv.nevada.edu (S.B.); fraga@unlv.nevada.edu (B.R.); lianne.barnes@unlv.edu (L.B.); sheikn1@unlv.nevada.edu (N.S.-Y.); villae12@unlv.nevada.edu (E.V.); wackens@unlv.nevada.edu (J.W.); 2 LGBTQIA+ Community Center of Southern Nevada (The Center), Las Vegas, NV 89101, USA; jwaldron@thecenterlv.org; 3 Centre for Addiction and Mental Health, Toronto, ON M5S 2S1, Canada

**Keywords:** COVID-19, digital technologies, pandemic, older men who have sex with men, resilience to HIV, social isolation

## Abstract

When the COVID-19 pandemic emerged in early 2020, not only did it abruptly impede the progress that was being made toward achieving global targets to end the HIV pandemic, but it also created significant impacts on the physical and mental health of middle-aged and older men who have sex with men living with HIV. Utilizing a qualitative, community-based participatory research approach, we conducted semi-structured, one-on-one interviews with 16 ethnoracially diverse, middle-aged and older men who have sex with men living with HIV residing in Southern Nevada, to examine the different ways the COVID-19 pandemic directly impacted their physical and mental health, and explore how they eventually coped and thrived during the peak of the crisis. Using thematic analysis to analyze our interview data, we identified three prominent themes: (1) challenges to obtaining credible health information, (2) the physical and mental health impacts of the COVID-19-pandemic-imposed social isolation, and (3) digital technologies and online connections for medical and social purposes. In this article, we extensively discuss these themes, the current discourse on these themes in academic literature, and how the perspectives, input, and lived experiences of our participants during the peak of the COVID-19 pandemic could be critical to addressing issues they had already been experiencing prior to the emergence of the pandemic in 2020, and just as importantly, helping us best prepare in stark anticipation of the next potentially devastating pandemic.

## 1. Introduction

In the last two decades, middle-aged and older men who have sex with men living with HIV (MSMLWH) have been a prominent focus of health and social science research due to their significant healthcare and social service needs associated with HIV-related premature aging, neurocognitive and mental health challenges, and syndemic comorbidities [1,2]. In general, as they age, MSMLWH and other people living with HIV (PLWH) have a higher likelihood of encountering physical health challenges associated with cardiovascular disease, hypertension, and diabetes mellitus [3]. Research in social sciences has documented that MSMLWH and other PLWH have higher rates of depression, anxiety, and substance use related to their lack of access to mental health resources compared to their heterosexual counterparts [4,5,6]. In light of these challenges, healthcare specialists and providers in social services have sought out solutions to manage these growing challenges. In an effort to address the needs of older MSMLWH and other PLWH, researchers have examined the potential of innovative and affordable health system models [7]. Other researchers have proposed the implementation of successful health system models that incorporate diverse and specialized medical fields (e.g., infectious diseases, neuropsychiatry, cardiology) to meet the complex physical health needs of older populations living with HIV [8]. Social services for older MSMLWH and other PLWH have begun incorporating specialized features of care in order to combat the unique challenges faced by older populations. Attention has been devoted to addressing factors associated with depression and anxiety, such as social isolation and lack of social support, which have successfully been targeted and reduced with the use of community-based interventions for older MSMLWH [9]. Brennan-Ing and colleagues [9] have documented how study participants who engaged in daily informal conversations amongst a social network of older MSMLWH reported a decrease in depressive symptoms following such an intervention. Related research has suggested that further investigation should be carried out to explore the impacts of the quality and different aspects of social support on HIV care outcomes [10].

By the end of the year 2014, nearly 75% of MSMLWH in the US have received some form of initial HIV care, but only about 58% have remained in continuous care [11]. Research has investigated factors that keep MSMLWH from receiving continuous care. Prominent factors have included institutional and structural barriers to access to care (e.g., HIV stigma, systemic racism, poverty, incarceration, and homelessness) [12], meager support from their own communities [13], and a lack of familiarity with potential resources [14]. In the past decade, these barriers have been critically reviewed by researchers to help meet the goal of ending HIV [14], and its long-lasting impacts as a global pandemic [15]. More recently, scholars have noted the importance of gaining and incorporating the perspectives and input of older MSMLWH and other PLWH while exploring technological innovations and enacting public policy related to HIV care as a means to achieving these goals [16].

With the emergence of the COVID-19 pandemic during the first quarter of 2020, the devastating clinical and social impacts of an unprecedented, new, worldwide public health emergency began to rapidly unfold [17]. The greater risks of contracting or experiencing complications from COVID-19 became potentially significant concerns for people with chronic health conditions, including MSMLWH and other PLWH [18]. For MSMLWH and other PLWH, considerable disruptions in the delivery of HIV care and programming due to shelter-in-place orders and other isolation guidelines and protocols became almost immediately apparent [19,20,21]. These included disruptions in HIV, CD4 cell count, and viral load testing [19]; HIV prevention and treatment services [20]; and HIV follow-up and continued care [19,20]. In addition to the impacts of COVID-19 on MSMLWH and other PLWH in terms of clinical risks and outcomes, the social impacts of the new pandemic related to stress and mental health; individual isolation and loneliness; food insecurity; housing instability; changes in sexual behavior; changes in substance use; low income and record unemployment; and racial and ethnic disparities, also came to light [22].

In the last three years, as a response to the public health emergency, a glut of research studies on the clinical and social impacts of COVID-19 on people living with or at risk of HIV was conducted in the US [18,21], and this trend was also reflected in research that was carried out around the world [20]. Across the globe, judicious and timely research was also conducted on the impacts of COVID-19 specifically on MSMLWH [20], as well as men who have sex with men at risk of HIV [21]. Although there were numerous studies that were conducted that predominantly focused on the COVID-19 impacts on youth and young adult men who have sex with men at risk of or living with HIV [19,20,21], it was clear that there was not as much research done that was explicitly focused on older PLWH [23], and to the best of our knowledge, none exclusively focused on older MSMLWH, the subpopulation that has historically been impacted by HIV the longest, and likely the subpopulation that has exhibited resilience to HIV the most [24].

In this article, we examine and discuss the impacts of COVID-19 on ethnoracially diverse, middle-aged and older MSMLWH during the peak of the COVID-19 pandemic based on the perspectives, input, and lived experiences of our relevant study participants from Southern Nevada. We also explore and discuss the ways our older MSMLWH participants coped and thrived, despite the overwhelming challenges they experienced during the crippling lockdowns and stay-at-home orders that were enforced during the COVID-19 pandemic that rapidly emerged in 2020.

## 2. Materials and Methods

### 2.1. Participants and Procedures

In collaboration with several lesbian, gay, bisexual, trans, and queer (LGBTQ) not-for-profit agencies, AIDS service organizations, and other community-based partners from our region, we purposively recruited relevant stakeholders to participate in our qualitative interviews. Prior to conducting our study, we developed a research protocol that was evaluated and ratified by our community partners, and subsequently approved by the Institutional Review Board (IRB) of the Office of Research Integrity of the University of Nevada, Las Vegas. Utilizing IRB-approved printed flyers that we posted on the premises of our community partner agencies and organizations, and recruitment messages that we made accessible through their various email listservs and websites, we recruited prospective participants using specific inclusion criteria. In order to take part in our study interviews, participants needed to be: (1) 40 years of age or older, (2) living with HIV for at least one year, (3) a resident of Southern Nevada, and (4) a man who self-identified as gay, bisexual, queer, or a man who has sex with other men. We carried out our study involving participants residing in both urban and rural communities with relatively limited access to physical and mental healthcare when compared to the national average [25]. We conducted our semi-structured, confidential, one-on-one interviews with middle-aged and older MSMLWH in Southern Nevada from January to April 2022 through Zoom, a video chat and conferencing platform. We continued to recruit and interview participants until data saturation for key themes was achieved (i.e., no new information relevant to the key themes emerged as additional interviews were conducted). Our 16 participants’ ages ranged from 41 to 68 years old, and their average age was 54. All our participants self-identified as gay, were receiving HIV care, and taking their prescribed antiretroviral therapy medications at the time of their interviews. In terms of race, our participants identified as White (50%, *n* = 8), Black (37.5%, *n* = 6), Middle Eastern (6.25%, *n* = 1), and Asian-Pacific Islander (6.25%, *n* = 1). We assigned each participant a pseudonym from the time of their interview, and we used their respective pseudonyms to identify them in this article. We compensated each participant with a $50 gift card for their time and effort to participate in our interviews.

### 2.2. Materials

Our research team and community partners contributed to the development of our IRB-approved interview guide. Our interview guide questions represented an exploration of various domains under the context of the COVID-19 pandemic (e.g., early experiences, relationships, healthcare access, and health literacy). These questions and domains were based on the discourse found in academic literature that focused on the wellbeing of older MSMLWH [2], and were subsequently reviewed and approved by community partners from Southern Nevada. We constructed broad questions to encourage participants to explore their general experiences during the COVID-19 pandemic, (e.g., “Could you please describe what your social relationships were like at the start of the pandemic?*”*), as well as prepared potential follow-up questions to further examine each participant’s specific responses to the broad questions. The interview questions were open ended, and the virtual interviews lasted between 40 and 60 min. With each participant’s consent, their interview was audio-recorded and transcribed verbatim to prepare the interview content for data analysis.

### 2.3. Analysis of Data

We analyzed our de-identified and transcribed qualitative data using thematic analysis [26]. Guided by the different phases described by Braun and Clarke [26], our project coordinator and two other members of the research team familiarized themselves with the first half of our entire dataset as initial coders by reviewing the transcripts, recording initial ideas, and re-reading the transcripts for greater clarity. Next, each of our three initial coders identified preliminary codes separately, and then gathered to compare and discuss their codes until they reached an agreement on the code definitions in preparation for the creation of a coding manual. Following this phase, the initial coders collated the preliminary codes into overarching themes using a color-organized coding manual to stimulate higher-order thinking skills and quickly achieve a comprehension of the codes and themes [27]. The color-organized coding manual was then utilized for the analysis of the remaining half of the data set by both the three initial coders and three other members of our research team who participated as additional coders. During the latter phase of the analysis, each of the six coders used the coding manual to continuously contribute to the development of the themes from the remainder of the dataset. All coders participated in regular meetings to maintain an ongoing discussion of each theme across the analysis of each interview transcript until the consensus was reached.

## 3. Results

In our qualitative interviews, participants shared their personal experiences during the COVID-19 pandemic, particularly the challenges they experienced as older MSMLWH, working hard to remain resilient at a time of shelter-in-place orders, mandated lockdowns, and fettered access to HIV care due to considerable disruptions in healthcare delivery and social services. Our participants also shared their experiences on how they received support, learned of new resources, and gained new perspectives that helped them cope and thrive despite the clinical and social impacts of the COVID-19 pandemic. From our thematic analysis of our interview transcript data, we identified three prominent themes: (1) challenges in obtaining credible health information, (2) the physical and mental health impacts of the COVID-19-pandemic-imposed social isolation, and (3) digital technologies and online connections for medical and social purposes.

### 3.1. Challenges in Obtaining Credible Health Information

The first theme that we identified in our analysis was the challenges that surfaced when our participants attempted to gain new knowledge regarding the risks and other matters related to their health as the COVID-19 pandemic emerged. While most participants expressed an awareness of the need to be proactive about finding reliable information about their health risks, some participants shared an increasing concern for the trustworthiness of the health-related news sources that were available to them and their experiences, while navigating possible misinformation. As older MSMLWH, many of them lived through long periods of time, some even decades, of worrying about obtaining the most reliable and current information to maintain their health and wellbeing. A common concern while trying to find information related to one’s personal health and how to maintain it, particularly during the COVID-19 pandemic, was the ability to determine the credibility of the source sharing the news and the veracity of the information they provided.

In light of these concerns, many participants reported heightened experiences of unpleasant emotions when seeking information about their health, such as uncertainty, doubt, confusion, distress, and feelings of mistrust toward health information sources. Specifically, this newly formed mistrust during the early period of the pandemic was directed at various information sources such as community members, healthcare agencies, and the US government. Tim (50 years old, Black), who also happened to be a long-term service provider in Southern Nevada, expressed:

As somebody who’s worked in healthcare for so long, it was like realizing that you’ve been lied to for the first time by somebody you never expected to lie or publicly post inaccurate information. So, I question everything now that comes up, even information from the Center for Disease Control and Prevention (CDC). I question the research now, more so than ever. Because they faltered. Um, I can’t trust that a two-year study means an X amount of confirmed results, statistically… how much of it was politically influenced at this point.

Participants noted how emerging information about COVID-19 was seen as critical to managing their health. However, the information that was delivered by their local healthcare and wider government agencies was either not always easily accessible, seemingly mismanaged, or at times, too confusing for readers to confidently comprehend. Peter (50 years old, Middle Eastern American) recalled one of his earliest experiences in 2020, “So I get this letter in the mail. I really didn’t understand it because they were using all these big words. So I called the nurse and I said, ‘What is this? Do I have it? Or do I not?’.”

In addition to their newly developed mistrust towards healthcare and government agencies, some participants also revealed that they believed the way information regarding HIV and COVID-19 was being spread among PLWH in the community was not so trustworthy. According to one participant, Doug (61 years old, White):

People are so easy to persuade today, and it’s all because of social media. It’s also because of the internet. People can post whatever they want—misinformation, conspiracy theories, who knows! You can’t believe everything that you read on the internet. Some of these people are so naïve and gullible because they don’t educate themselves and verify information. They let some other person dictate their belief system. That’s what we’re dealing with in our country today, in our world….and it’s sad. It’s very sad. I think our world has changed tremendously, forever.

Participants expressed concern for the credibility of information shared by some HIV service providers from Southern Nevada. Some participants pointed to the spread of word-of-mouth news as a source of uncertainty, as they tried to distinguish credible from unreliable health information. Joey (46 years old, Asian American) explained how potentially false information was spreading and consequently creating a false sense of security among older MSMLWH in the community:

There were rumors or speculations going around that our [HIV] medications were keeping us from getting really violently sick [from COVID-19]. Like, everybody else was dying from it, but people with HIV on meds were doing pretty good. I don’t know if that was ever tested or confirmed. But I think that was just speculation amongst members of our community.

Overall, our participants’ uncertainty while determining credible health information that was presented to them in the previous two years elicited strong negative emotions. Concerned and confused participants described the impacts of not understanding the realities of the COVID-19 pandemic in relation to their own health needs. Jonathan (64 years old, White) shared his growing fears as the COVID-19 pandemic persisted:

It was frightening too! In the beginning, we didn’t know what was gonna happen. We didn’t know what we were dealing with, and I was afraid to go out anywhere. I was afraid to talk to people even about my increasing fears for my health.

Tim (50 years old, Black) echoed the trepidation that Jonathan described:

I was scared and I ended up telling someone that I was really nervous because I’m immunocompromised. This is not a fun place to be in, and I was nervous…I had fevers that were pushing a hundred and four for almost like six or seven days, and just couldn’t get it broken. I was convinced that because of HIV, I was not gonna make it. I mean, I was scared as hell!

Some participants found it important to clarify that they were fully aware that online and word-of-mouth misinformation had already been major problems in recent history even before the emergence of COVID-19. However, they emphasized that the pandemic and the disruptions to HIV-related healthcare delivery and social services definitely made online and word-of-mouth misinformation even bigger issues, particularly concerning information they needed to mitigate their risks and stay healthy. At the peak of the COVID-19 pandemic, as clinics, community health centers, and community-based organizations shut down, the only sources of information many of them could access were the internet and a community of anxious PLWH sharing unverified health news with each other through remote or virtual communication.

### 3.2. The Physical and Mental Health Impacts of the COVID-19-Pandemic-Imposed Social Isolation

The second theme that we identified from our interviews was the impact of social isolation on the physical and mental health of middle-aged and older MSMLWH. While several participants candidly shared that both their physical and mental health were impacted by the new pandemic, most participants revealed that it was their mental health that significantly suffered as a direct result of the social isolation brought about by the COVID-19 social distancing protocols, which were imposed at the beginning of 2020. Despite the availability of the internet and other digital technologies to stay socially connected, multiple participants consistently referred to the adverse mental health outcomes of social isolation during their interviews. Many participants described the social isolation they experienced as something very irregular in terms of their typical lifestyles, especially being quarantined at home by themselves or being limited to the physical presence of one other person from their social circle. As middle-aged and older MSMLWH, some participants revealed that they already had a diminishing social circle, having lost family and friends over the years due to rejection or HIV-related comorbidities. Laurence (56 years old, Black) recounted his experiences as almost everything in the region was shutting down in a matter of weeks:

I’ve felt very socially isolated. I’ve always relied on my job to provide a social atmosphere for me just because I don’t have any kids. I love my job and tend to throw myself into it… having to isolate and work from home, or even not being able to see clients face to face, and instead do Zoom meetings…it was hard to make connections with people.

Participants described these moments of social isolation as times that led up to instances of depression, heightened levels of stress, dissociation from life, urges to relapse with problematic substance use, feelings of anger, and overall moments of darkness. David (41 years old, White) was quite explicit about his own challenges:

I’ve noticed it in my own personal recovery from substance use. My urges to use have gone up. Just overall stressors. Some days, I kind of get the feeling like, “Jeezus, if this our new normal, then what’s the fucking point of living?”. I’ve had a couple of horrible thoughts during the past two years, where it’s like, “Why don’t I just stop taking my medication and let nature run its course?”. I’ve never had thoughts like that before. It’s more than just the isolation. It’s also the loneliness and how disconnected everything is. They all have a huge impact on your mental health.

Participants also shared how feelings of dissociation and heightened levels of stress ultimately influenced their physical health. For some participants, the detrimental impacts of their heightened levels of stress became apparent in their laboratory results. Bobby (56 years old, Black) recognized the changes associated with the stress he experienced from being socially isolated:

The stress from this pandemic was noticeable in my lab work. There was a drop in my CD4 count. My physician noticed it. He was like, “See this, these are indicators of too much stress. I’m seeing them across the board with my patients during the pandemic.” It was just stressful. So, it’s actually causing my CD4 count to go lower. So, it makes sense, the more stressed you are, the more likely you are to get sick because it has an impact on your immune system. But to see it, like to actually see it in black and white with my numbers and my name…it was like, very eye opening.

Participants also shared instances of feeling unmotivated, which influenced their decisions and behaviors, such as not showing up for medical appointments or refraining from taking their prescribed medications. John (41 years old, Black) revealed how a loss of motivation could negatively affect one’s desire to maintain self-care, “I think I stopped caring there for a little while. I got super depressed, stopped taking my medication for a bit, and just kind of went into a hole.”

In an attempt to combat social isolation and increase feelings of empowerment, a few participants shared how they searched for meaningful opportunities to contribute safely to their community by educating others or sharing tangible resources (i.e., creating and offering free masks). William (54 years old, White) reported how he wanted to try to turn things around by purposefully connecting with others virtually:

I started educating people, my coworkers around me, who had as much concern as I did. I encouraged people to observe hand washing and wear masks, which helped them not feel vulnerable. It was like, when you can help other people feel safe through a pandemic, it’s very empowering to know that you can do something useful even though you have no control over this coronavirus.

Lastly, despite the physical and mental health setbacks brought about by social isolation, participants shared the importance of remaining hopeful for the future, viewing challenges from a positive mindset, and cultivating lighthearted virtual spaces. For instance, participants had described the importance of engaging proactively in online support groups as a means to combat feelings of isolation. In his interview, Matthew (61 years old, White) shared, “I no longer have the same negative thoughts I had when the COVID-19 pandemic started. But, I still feel that if I get it, I’m not gonna survive it. That’s why I’m doing virtual support groups to feel less isolated.”

### 3.3. Digital Technologies and Online Connections for Medical and Social Purposes

The last theme that we identified was the use of digital technologies and online connections for the purposes of meeting the participants’ medical and social needs. Interestingly, we identified digital technologies and online connections in most of the interviews as both a challenge as well as a facilitator to addressing their medical needs and maintaining their social connections. The use of digital technologies (i.e., the internet, social media, social networking and messaging apps) and other online forms of personal, group, or community connection such as Zoom, were frequently reported by our participants as they described how they accessed their HIV care, and health and support services during the COVID-19 pandemic.

Participants relied on digital technologies and other online connections as a means to access primary care, medications, diagnostic and medical laboratories, recovery programs, and mental health services. Since most communications and networking eventually became virtual, it did not take long for our participants to identify the challenges they needed to face as they struggled to obtain their HIV care and support services. Multiple participants noted how mentally exhausted they felt after a prolonged amount of screen time due to online-video-formatted healthcare meetings. Specifically, participants reported experiences of “Zoom fatigue” as a challenge when accessing healthcare through online services, and in response, advocated for returning to in-person services or taking breaks between sessions. After several months of virtual interactions, Keith (52 years old, Black) described his personal experiences with “Zoom fatigue”:

Everything has been going virtual and telehealth. It becomes kind of mundane. After a while, you get Zoom fatigue like, “Oh good Lord, not another [therapy session or support group] Zoom meeting”. All you’ve been doing the entire week is Zoom, Zoom, Zoom. It’s like your brain kind of turns off from Zoom. Like you wanna get out of the house a little bit, go see something, and be around people.

Some participants critiqued the level of online accessibility from healthcare providers. They reported challenges that ranged from healthcare providers not having any online or remote options at the start of the COVID-19 pandemic to the notable delays in online or remote communication when it was made available. Jonathan (64 years old, White) recalled the huge technological shift that was needed at the start of the COVID-19 pandemic, and how older MSMLWH, such as himself, did not know what was going to happen with the support they were used to receiving in person:

Initially, the therapists weren’t seeing people face-to-face, and they weren’t doing any meetings online [over Zoom] or on the phone either. So I just kind of stopped getting therapy. I think everybody was really struggling too. I mean, we have all this great technology, but we never used it as much as we have in the last couple of years. So, there was a big communication gap for a while. Sometimes you’d call, you’d leave a number, and call backs were…well, people wouldn’t call back.

Commonly reported challenges that participants encountered while attempting to maintain social relationships through online engagement were related to their difficulties in achieving more intimate social connections. Participants reported that the capacity to foster intimate and personal conversations or read body language within online spaces was quite limited compared to in-person interactions. Although many participants reported the advantages of using digital technologies and going online to seek social relationships, some participants preferred and opted for in-person sessions to build deeper social connections. Mickey (58 years old, White) explained his frustration with video chat conversations:

Over Zoom, you don’t really get to know people. You never feel like a really great connection. Like if I’m here, I can read some body language…there’s more involvement in terms of connection when you’re sitting in front of me. When were just looking at each other on Zoom, I don’t really feel a connection with you, and therefore, I can’t dig at those things that I’m interested in or that you’re interested in. So it seems very much just on the surface. It doesn’t get very intimate on Zoom, and in fact, in a lot of Zoom meetings, like half the people turn off their cameras.

In light of the challenges related to relying on digital technologies and other online services during the COVID-19 pandemic, participants also noted the very important benefits related to accessing healthcare virtually or engaging in social activities online. Despite the challenges associated with the limitations of virtual meetings for medical or therapy consults and follow-ups, many participants reported that their more recent experiences with using digital technologies were very beneficial due to the convenience of accessing and managing healthcare appointments, the prompt responses from healthcare and service providers, and an increased sense of autonomy in securing medical supplies via mobile phone applications or self-service mobile medical dispensers (i.e., vending machines). After further reflection on their evolving circumstances and eventually getting over their growing frustration with “Zoom fatigue”, Keith (52 years old, Black) reported that he began to recognize how digital technologies and other online connections were able to facilitate his efforts to successfully address his medical needs:

Actually, I soon realized that these virtual options make it easier for me to keep track of information and everything else, and not to have to travel so much all around anymore. The services I need are all over Downtown, so I don’t have to commute from place to place. So now, if I do have a doctor’s appointment, and they do have telehealth, I’d prefer that. It’s been helpful because it helps me keep up with my appointments a bit better, and I get to stay in the HIV care continuum with less hassle.

Beyond utilizing them for health-information-related and medical purposes, participants also leveraged the potential of digital technologies and online services to maintain established social relationships and develop new connections. Participants reported how they were able to join more support groups and explore new connections virtually that would not have been possible prior to the COVID-19 pandemic. Since the introduction of telehealth and virtual services and programs during the pandemic, not only did participants report that pre-COVID-19 support groups within the community became more easily accessible online, but participants also shared how novel and newly offered support groups became easier to join due to their online accessibility. Online engagement in these new support groups were reported as beneficial to bolstering social activity and combating social isolation. Burt (68 years old, Black) commented on the additional benefits to having access to virtual communications and connections:

I’m not a bar person that goes where people go. When they’re online, you start talking to different people in [support] groups. You start getting closer to them, and your [group] associations later become more like friendships. This is something I didn’t have before COVID-19 hit. Occasionally, the online associates, we met in person. I could, in fact, be more socially active now than I ever was prior to the pandemic.

As they became more adjusted to the impacts of the COVID-19 pandemic, many of our participants had become less reluctant and more comfortable to acknowledge the positive roles that digital technologies and online connections played in helping them overcome the struggles they experienced due to their challenges in obtaining credible health information, social isolation, and the statewide disruptions in healthcare delivery and social services, which were brought on or aggravated by the pandemic.

## 4. Discussion

Since the start of the HIV crisis, tens of millions of PLWH have died from AIDS [28], and today, MSMLWH (particularly older MSMLWH) in the US and around the world continue to struggle with obstacles to maintaining their health and wellbeing as a result of the persistence and evolution of the HIV pandemic [12]. Although several other pandemics, due to the spread of different deadly viruses (i.e., SARS, H1N1, Ebola, Zika), have emerged in the 21st century, none have caused so many tragic deaths globally, crippled the world economy, and adversely impacted those living with HIV with significant disruptions in healthcare delivery and social services as much as the COVID-19 pandemic [29,30,31]. Our study explored the lived experiences of ethnoracially diverse, middle-aged and older MSMLWH in Southern Nevada during the COVID-19 crisis; investigated how they were affected by the COVID-19-pandemic-imposed social isolation; and examined how they coped and thrived as they went through the impacts of the COVID-19 pandemic upon a decades-long HIV pandemic that some of them have resiliently endured for over forty years.

### 4.1. Misinformation and Challenges in Obtaining Credible Health Information

In terms of its associated mortality rate around the world, its devastating impact on the global economy, the significant disruptions in healthcare delivery and social services it has caused, and its adverse effects on the health and wellbeing of PLWH, the COVID-19 pandemic has set an unprecedented record during the contemporary age of digital technologies. Although past pandemics have similarly impacted countless lives across the globe, the constant mass and social media coverage of the COVID-19 crisis had placed it at the forefront of millions of people’s minds and hearts, and as the backdrop for contentious political battle, especially in the US [32]. In the 21st century, the ubiquity and popularity of various virtual and online platforms has meant that the public is no longer merely passively consuming inaccuracies and fear-mongering falsehoods, but is also actively contributing to the perpetuation of unreliable and even dangerous information [33].

During their interviews, our participants not only reported how difficult it was for them to obtain credible health information, particularly information relevant to them as middle-aged and older MSMLWH and their specific risks during the COVID-19 pandemic, but they also expressed their frustration with having to contend with retractions and conflicting information from sources they believed for a long time as reliable and credible. Case in point, some of our participants felt disheartened when the CDC repeatedly reversed itself and made three distinct revisions on COVID-19 information or guidelines that they published on their website since May 2020 [34].

Our participants’ narratives notably echoed previously documented frustrations of other older MSMLWH during the COVID-19 pandemic [35], underscoring the salience of their challenges to obtaining credible health information that was vital to them as a subpopulation that is potentially at a greater risk of complications if they contracted COVID-19. Related to these findings, researchers have suggested the importance of fostering media and digital literacy skills, particularly among vulnerable populations, in response to the rapid spread of misinformation online and in communities (i.e., infodemics) [36]. While previous research has suggested that rural populations may have limited media and digital literacy skills that need to be substantially improved [37], our study emphasizes the importance of investing in the media and digital literacy skills of middle-aged and older MSMLWH in both urban and rural contexts.

### 4.2. The COVID-19-Pandemic-Imposed Social Isolation as a Key Health Risk

Older PLWH are more likely to live alone and have been found to have considerably lower scores on the 18-item Lubben Social Network Scale (LSNS-18) than younger PLWH [38], which has prospectively left them at a greater risk for social isolation. Older MSMLWH in the US, in particular, have often lived with HIV longer than other subpopulations with the virus, and have been more likely to confront the stress of managing more complex HIV disease than their younger counterparts [39]. Social isolation has already been found to be a key health risk among older MSMLWH [40]. Older MSMLWH have historically already had to withstand the adverse health risks brought about by social isolation due to HIV stigma and other causes [2], and as our study findings revealed, the COVID-19 pandemic brought about new and confounding struggles related to social isolation that our participants had to endure.

### 4.3. Maintaining the HIV Response Using Digital Technologies and Online Connections

Most of our participants recognized that digital technologies and online connections could be key solutions to addressing the struggles they had as middle-aged and older MSMLWH that were present even before the emergence of the COVID-19 pandemic (e.g., accessing credible HIV information, care, social support, and personal connections in their region where healthcare and social services are accessible only to people with a reliable means of transportation). Overall, the perspectives and lived experiences of our participants confirmed the inherent potential and possibly prodigious roles digital technologies and online connections could play in maintaining the HIV response in the future, and optimistically, ending the HIV pandemic.

#### 4.3.1. Utilizing Digital Technologies and Online Connections to Combat Misinformation

Even before the COVID-19 pandemic began, most of our participants had already been aware that the spread of rampant misinformation perpetuated by mass and social media had been a growing problem in the last decade. Researchers and professionals likewise had been aware of this problem, and scholarly work had already begun to reflect the emerging concern for the adverse effects of rampant misinformation on the health and wellbeing of the public, particularly the underserved. Despite the academic and public acknowledgement and understanding that digital technologies and online connections were a major source and part of this problem, scholars have started to assert that digital technologies and online connections could potentially be an important part of the solution to addressing rampant health and political misinformation [41,42,43], especially during times of almost uncontrollable health crises, such as the COVID-19 pandemic [44]. Some scholars have provided individual-level recommendations for public consumers to consider (e.g., learn about search engine ranking, read before sharing, fact-check, seek information beyond your filter bubble, use verification and educational tools) and consequently practice [41], while other scholars have presented recommendations that are more applicable to implement at the community level (e.g., perform network content validation, conduct media literacy methodologies and workshops, promote public information campaigns, foster organizational cooperation). Nevertheless, other scholars have conducted research that has generated findings to aid policymakers in making evidence-based decisions to combat the spread of misinformation, including crucial themes for public-policy-making to consider or implement, such as the creation of trusted networks of experts and collaborators, the provision of access to visualizations of data at different levels of granularity, and the delivery of innovative ways to increase the transparency and explainability of flagged misinformative content [44].

Although the usefulness of social media, digital technologies, and online connections has been questioned by skeptics [45], many HIV researchers have documented that social media, digital technologies, and online connections are not only effective for scaling up credible information-sharing [46,47,48,49,50], but also very useful for combating misinformation and countering stigmatizing narratives [43,51]. In fact, digital technologies and online connections have been reported to be effective tools for promoting evidence-based continuing education [51,52,53], clinical and study recruitment [48,53], HIV prevention and intervention campaigns [46,47,51,53], specialized health information [50,53], and stakeholder behavior change [49,50,53,54].

#### 4.3.2. Using Digital Technologies and Online Connections to Address Social Isolation

In the wake of the distressing impacts of the social isolation brought about by the COVID-19 pandemic on vulnerable populations, the increasing public health concern for the welfare of middle-aged and older adults and their health had resulted in an upsurge of studies to uncover ways to deal with the COVID-19-pandemic-imposed social isolation among the elderly, including research exploring the inherent potential and critical roles of digital technologies and online connections to overcome such social isolation [55,56,57,58]. Two scoping reviews on the use, effectiveness, and value of interventions using digital technologies and online connections in reducing social isolation among older adults had been conducted in the last year, both of which revealed the positive effects of technology on alleviating social isolation and loneliness [55], and the essential need to understand the degree to which technological interventions could continue to positively influence social isolation and loneliness among older adults, even beyond the pandemic [58]. One timely study, in particular, had highlighted the urgent need to invest in the development of appropriate digital technologies to reduce the impacts of social isolation during the COVID-19 pandemic and their associated long-term costs to the healthcare system [57].

#### 4.3.3. Maximizing Digital Technologies and Online Connections to Sustain HIV Care

In addition to the valuable roles of digital technologies and online connections to combat misinformation, disseminate accurate and credible health information, and address the impacts of the COVID-19-pandemic-imposed social isolation, research during the peak of the COVID-19 pandemic has shown that digital technologies and online connections could potentially be used to sustain HIV care even more directly by focusing on and preserving critical services and programs [59,60]. As circumstances became more dire, several researchers and advocates had called for efforts to ensure that access to HIV services not only be maintained, but also protected and expanded during the COVID-19 pandemic [61,62]. Indeed, notable HIV programs and AIDS service organizations had not only kept their services going despite the difficulties they needed to overcome with statewide shutdowns and limited resources [31], but they had also expanded their services (e.g., antiretroviral therapy distribution and dispensing) accordingly in response to the crisis [59]. Other researchers had additionally called for expedited HIV-differentiated service delivery during the COVID-19 pandemic [63], leading other researchers to look and turn to the promise of digital technologies and online connections to bring timely and more contextualized solutions centered around the needs of PLWH, including older MSMLWH.

Even before the emergence of the COVID-19 pandemic, researchers had already begun to explore the inherent potential and critical roles that digital technologies and online connections could play to improve HIV care, programming, and prevention interventions [64,65,66]. Numerous studies have shown that interventions using digital technologies such as eHealth (i.e., electronic health services offered through the internet), mHealth (i.e., mobile health services delivered telephonically, as full-blown applications (apps), or via short message service (SMS)/texting on mobile phones and other devices), and telehealth (i.e., health-related information and services provided over electromagnetic, radio, wire, and optical systems, and other digitized telecommunication technologies including video conferencing or video chat), have emerged as a promising approach to supporting the ongoing creation of diverse opportunities for health promotion along the HIV care and prevention continuum, ensuring linkage and retention in HIV care, and fostering lifelong engagement in care after diagnosis [64,65,66]. These digital technologies and online connections have been increasingly utilized and evaluated over the last decade in large clinical settings, remote and rural regions, and other resource-poor settings [67], exhibiting much promise as practical solutions to persistent issues commonly found in the delivery of HIV care.

Locally, similar efforts had been implemented by healthcare and service providers, researchers, and relevant stakeholders in the Southern Nevada HIV sector. Through Zoom video conferencing, healthcare and service providers were able to help MSMLWH distinguish credible health information from false reports and fake news, keep them engaged in their support groups or introduce them to new ones to overcome social isolation, and ensure that they remained in the HIV care continuum throughout the pandemic. SMS/text messaging allowed for our participants to stay in touch with their providers and peers from the community, and became an important and inexpensive tool for receiving reminders for consultation and follow-up appointments, as well as scheduled laboratory exams. Access to the internet even gave them opportunities to participate in several study surveys and interviews, which provided them meaningful outlets to express their sentiments and perspectives based on their lived experiences. By the time the adverse impacts of the COVID-19 pandemic began to abate, our participants started to recognize and appreciate digital technologies and online connections as tools that could be utilized and harnessed even after the end of the pandemic.

As residents of Southern Nevada, our participants had always had to contend with their predicament that despite the fact that they had numerous and valuable resources in their region in terms of HIV programs and services, these HIV programs and services were not particularly easy to access as the HIV clinics and community health centers, AIDS service organizations and LGBTQ not-for-profit agencies, and important service-related events were spread out across the region. Based on our participants’ accounts, using digital technologies and online connections as a permanent alternative to accessing HIV care and support services in person serendipitously emerged as an extremely viable and even attractive option for them and their providers to seriously consider post-pandemic.

## 5. Strengths and Limitations of the Study

Our study adds important knowledge to the relatively sparse existing academic literature that focuses on the ongoing barriers and facilitators to building resilience to HIV that middle-aged and older MSMLWH experience as they age. In particular, the context of our study is distinct as it focused on the unique perspectives and lived experiences of middle-aged and older MSMLWH who resided in the sprawling region of Southern Nevada during the COVID-19 pandemic. When the COVID-19 pandemic emerged, we encountered significant obstacles in engaging and recruiting prospective study participants at the beginning of our research process, and thus, we relied heavily on the support of our multiple community partners who helped us gain substantial interest and involvement from relevant stakeholders in Southern Nevada. The main strength of our study was largely due to the candid and articulate responses, and rich and explicit data that our 16 participants provided.

It is important to also acknowledge our study’s limitations. Despite receiving a lot of support from LGBTQ not-for-profit agencies, AIDS service organizations, private clinics, and community health centers from Southern Nevada in terms of advertising our study and recruiting eligible participants, our study fell short of gaining perspectives and input from a more diverse and inclusive set of interviewees. Although our study aimed to involve a greater range of participants in terms of their ethnoracial backgrounds, as well as how participants self-identified in terms of their gender identity and sexual orientation (i.e., cis or trans; gay, bisexual, queer, or simply, as a man who has sex with other men), our participants were not as diverse in these terms as we hoped. The vast majority of our participants were either White or Black, and all of our participants identified as cis and gay. Inasmuch as our interviews provided an abundance of information from a very specific population of participants, the notion of absolute data saturation from our 16 interviews cannot be entirely ensured. Whether or not more interviews with participants under our current restrictive inclusion criteria would have proved effective in truly providing additional significant data remains a question. It is also important to note that the findings of our study, particularly the perspectives and lived experiences of our participants, are applicable specifically to the context of Southern Nevada, and potentially to other regions with similarly sprawling landscapes in North America. However, we are hopeful that the lessons and key points we learned from our study could be useful to more and larger contexts that could be examined in future research.

## 6. Conclusions

One lesson and key point that we learned from the perspectives and lived experiences of our participants in our study is that the increased use of digital technologies and online connections during the COVID-19 pandemic made them realize the potential role and value of such technologies and connections to address issues related to accessing their HIV-related healthcare and social services, which they struggled with even before the COVID-19 pandemic emerged. A second lesson and key point that we learned is that with an expansion and greater development of the use of digital technologies and online connections to provide more innovative ways for middle-aged and older MSMLWH to access their HIV-related healthcare and social services, we could also potentially be better prepared as a society for other forms of crises in the future. While the interest, trust, and public support for the use of digital technologies and online connections is still at a relatively high level after the COVID-19 pandemic, private and public HIV sector organizations and entities could prospectively take advantage of this momentum in the near future by prioritizing digital technological innovations in their own settings, not only to improve access to credible health information, provide support against social isolation, and deliver essential HIV programs and services for older MSMLWH and other PLWH, but also best prepare in stark anticipation of the next potentially devastating pandemic.

## Data Availability

Data is unavailable due to privacy and ethical restrictions.
